# Editorial: Emotions and emotional interplay within and between species: A “one welfare” perspective

**DOI:** 10.3389/fvets.2022.1011214

**Published:** 2022-09-05

**Authors:** Paolo Baragli, Jenny Yngvesson, Claudio Gentili, Antonio Lanata

**Affiliations:** ^1^Department of Veterinary Sciences, University of Pisa, Pisa, Italy; ^2^Bioengineering and Robotics Research Centre “E. Piaggio”, University of Pisa, Pisa, Italy; ^3^Department of Animal Environment and Health, Faculty of Veterinary Medicine and Animal Science, Swedish University of Agricultural Sciences, Skara, Sweden; ^4^Department of General Psychology, University of Padova, Padova, Italy; ^5^Department of Information Engineering, University of Florence, Florence, Italy

**Keywords:** animals, human, one welfare, holistic, interdisciplinary approach, emotion

Finding a standard interdisciplinary definition of emotion is challenging, but we affirm that “an emotion is an internal process triggered by specific stimuli relevant to the subject.” Moreover, following the well-known Circumplex Model of Affects (CMA) (1), an emotional reaction can be interpreted as a point in a multidimensional space where each dimension is in charge of representing a specific reaction of the organism, such as pleasantness, arousal, or sadness. Of note, the most applied CMA model in research on animal emotions is the bi-axial framework which considers only the valence (positive or negative) and arousal (high or low) axes (2).

It is also worth noting that, until recently, research has primarily focused on negative emotions; however, research on positive emotional states is increasing (3, 4).

An emotional reaction depends on several factors that can act at both internal and external levels on the individual subject, provoking physiological and behavioral responses. However, scientific evidence has highlighted to what extent emotional response is strongly influenced by temporal (life experiences, which are layered over time) and spatial (events acting on the animal at a precise moment) stimuli external to the animal. These factors affect individuality, which has, at its very base, personality traits and genetic components. In summary, an emotional process induces physiological and behavioral changes modulated by the individual's subjectivity, with experience and the succession of environmental events in everyday life which become crucial factors in emotional feeling and, therefore, the affective state (5).

However, this view of emotions based exclusively on an individual's reactions is a limiting factor. It is, therefore, fundamental to translate what happens within the individual into the social sphere; this is because individuals are not closed boxes; rather, they engage in plenty of interactions with others of the same or different species. Thus, social relationship should be included as crucial factors affecting the rise and expression of emotions. This is particularly true for highly social species, such as animals used in livestock production (e.g., cattle, pigs, sheep, poultry, and goats) and in social and recreational activities with humans (e.g., dogs and horses).

Broadly, social exchanges can be a source of fear and anxiety for animals. However, as reported in pigs, the social environment can enable the implementation of reconciliation and affiliation strategies that act as a buffer against anxiety (Norscia et al.). Furthermore, normal social relationships can help animals manage and modulate their emotional response to better cope with environmental challenges (Norscia et al.).

The results obtained in pigs could be linked to the peculiarity of sharing emotions in a social context. In fact, emotions represent a powerful means of communication between individuals of the same—but also of a different—species (6), including the relationship between animals and human beings.

Indeed, sharing emotions even with individuals of different species seems to have common traits that have developed over the course of evolution, which is why emotional exchanges in inter- and intraspecific relationships leads to fitness advantages (6, 7).

Panksepp et al. (8) found that primary emotions are shared by all mammals, including humans, and originate in the same subcortical regions of the brain. Moreover, the basic physiological state of primary emotional reactions is mainly determined by “feeling safe.” Therefore, peculiarities of emotion are common to all mammals, and they are a crucial component of the evolutionary adaptation of an animal species facing the challenges of a changing environment.

Approaching these studies with a broad multidisciplinary and interspecific view could provide an exhaustive, helpful overview of what happens between animals and between animals and humans when a relationship, and thus an emotional interplay, is established (Leconstant and Spitz).

This phenomenon is particularly evident, for example, between humans and dogs. The emotional component of the relationship between these two species is regulated, among other things, by shared daily activity (Väätäjä et al.). Similarly, between horses and humans, the quality of the daily relationship influences animals' emotions and affective state (9).

Moreover, measuring and understanding animals' emotions is challenging since behavioral and physiological responses are not systematically correlated; rather, they can vary, with a specific physiological reaction that can be linked to different behaviors, according to the abovementioned features [individual, environment, and experience; see (10) for an example in horses]. For instance, high sympathetic activity can be linked to escape, freezing, or other behaviors during a fear reaction.

In recent decades, bioengineering solutions have been implemented in the human field to individually investigate the two channels (behavioral and physiological changes) when an emotion arises (11). These innovative solutions enable a rebuilding of the emotional outcomes by combining different behaviors with different physiological reactions. Along with specific solutions for measuring physiology and behaviors, one of the most recent research challenges is the application of artificial intelligence and machine learning approaches to animal emotions (Neethirajan et al.). This could provide innovative tools to automate emotional assessments: for instance, in livestock, where the investigation at the individual level is not feasible or, when it is feasible, could lead to misleading results due to the influence of the social environment or to the limits of caretakers in detecting animals' affective states and emotions. The strength of automatized emotional recognition is that it could register the emotions of individuals in large groups.

It is worth noting that animals' lack of verbal communication about their feelings makes any emotional investigation complex (7). However, the importance of social relationships and emotions is highly relevant in animal welfare research (5), keeping in mind that animals' vocalization signals are part of the emotional response (12).

A One-Welfare perspective suggests that understanding the mechanisms by which emotions arise within a subject and are transmitted among subjects is essential for acquiring information regarding an animal's affective state. This aspect becomes even more relevant in animal husbandry or animal assisted interventions, where human presence is a binding condition for the animal.

Therefore, scientific research should develop new interdisciplinary paradigms and methods. That could suggest applying a holistic approach through monitoring indirect emotional measures, environmental factors, and/or social variables, and employing artificial intelligence solutions. This could enable effective recognition and, thereby, the classification of emotions and changes in animals' affective states, thus helping breeders, caretakers and owners as they implement strategies to modify the environment and animal management.

## Author contributions

All authors listed have made a substantial, direct, and intellectual contribution to the work and approved it for publication.

## Conflict of interest

The authors declare that the research was conducted in the absence of any commercial or financial relationships that could be construed as a potential conflict of interest.

## Publisher's note

All claims expressed in this article are solely those of the authors and do not necessarily represent those of their affiliated organizations, or those of the publisher, the editors and the reviewers. Any product that may be evaluated in this article, or claim that may be made by its manufacturer, is not guaranteed or endorsed by the publisher.
